# Hyper crosslinked polymer supported NHC stabilized gold nanoparticles with excellent catalytic performance in flow processes†

**DOI:** 10.1039/d2na00799a

**Published:** 2022-11-21

**Authors:** Constantin Eisen, Lingcong Ge, Elena Santini, Jia Min Chin, Robert T. Woodward, Michael R. Reithofer

**Affiliations:** a Institute of Inorganic Chemistry, Faculty of Chemistry, University of Vienna Währinger Straße 42 1090 Vienna Austria michael.reithofer@univie.ac.at; b Institute of Material Chemistry and Research, Faculty of Chemistry, University of Vienna Währinger Straße 42 1090 Vienna Austria robert.woodward@univie.ac.at; c Institute of Inorganic Chemistry – Functional Materials, University of Vienna Währinger Straße 42 1090 Vienna Austria jiamin.chin@univie.ac.at

## Abstract

Highly active and selective heterogeneous catalysis driven by metallic nanoparticles relies on a high degree of stabilization of such nanomaterials facilitated by strong surface ligands or deposition on solid supports. In order to tackle these challenges, N-heterocyclic carbene stabilized gold nanoparticles (NHC@AuNPs) emerged as promising heterogeneous catalysts. Despite the high degree of stabilization obtained by NHCs as surface ligands, NHC@AuNPs still need to be loaded on support structures to obtain easily recyclable and reliable heterogeneous catalysts. Therefore, the combination of properties obtained by NHCs and support structures as NHC bearing “functional supports” for the stabilization of AuNPs is desirable. Here, we report the synthesis of hyper-crosslinked polymers containing benzimidazolium as NHC precursors to stabilize AuNPs. Following the successful synthesis of hyper-crosslinked polymers (HCP), a two-step procedure was developed to obtain HCP·NHC@AuNPs. Detailed characterization not only revealed the successful NHC formation but also proved that the NHC functions as a stabilizer to the AuNPs in the porous polymer network. Finally, HCP·NHC@AuNPs were evaluated in the catalytic decomposition of 4-nitrophenol. In batch reactions, a conversion of greater than 99% could be achieved in as little as 90 s. To further evaluate the catalytic capability of HCP·NHC@AuNP, the catalytic decomposition of 4-nitrophenol was also performed in a flow setup. Here the catalyst not only showed excellent catalytic conversion but also exceptional recyclability while maintaining the catalytic performance.

## Abstract 2

Gold nanoparticles stabilized in a HCP·NHC network showing excellent catalytic activity in flow application.

## Graphical abstract

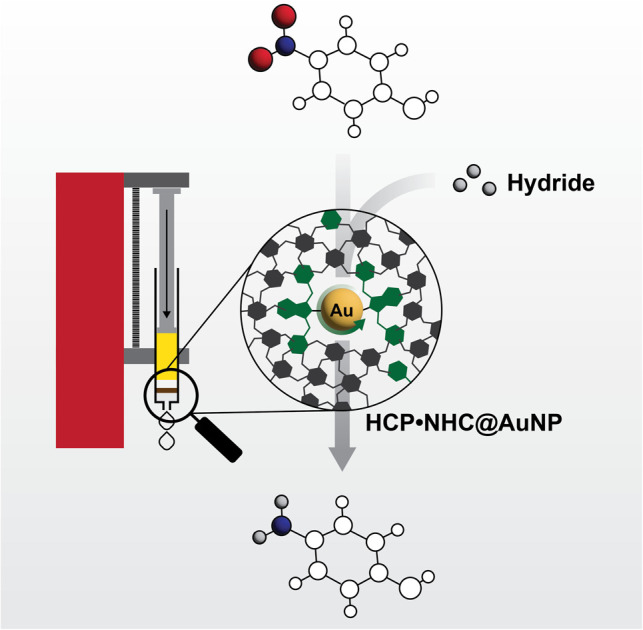

## Introduction

**Fig. 1:**
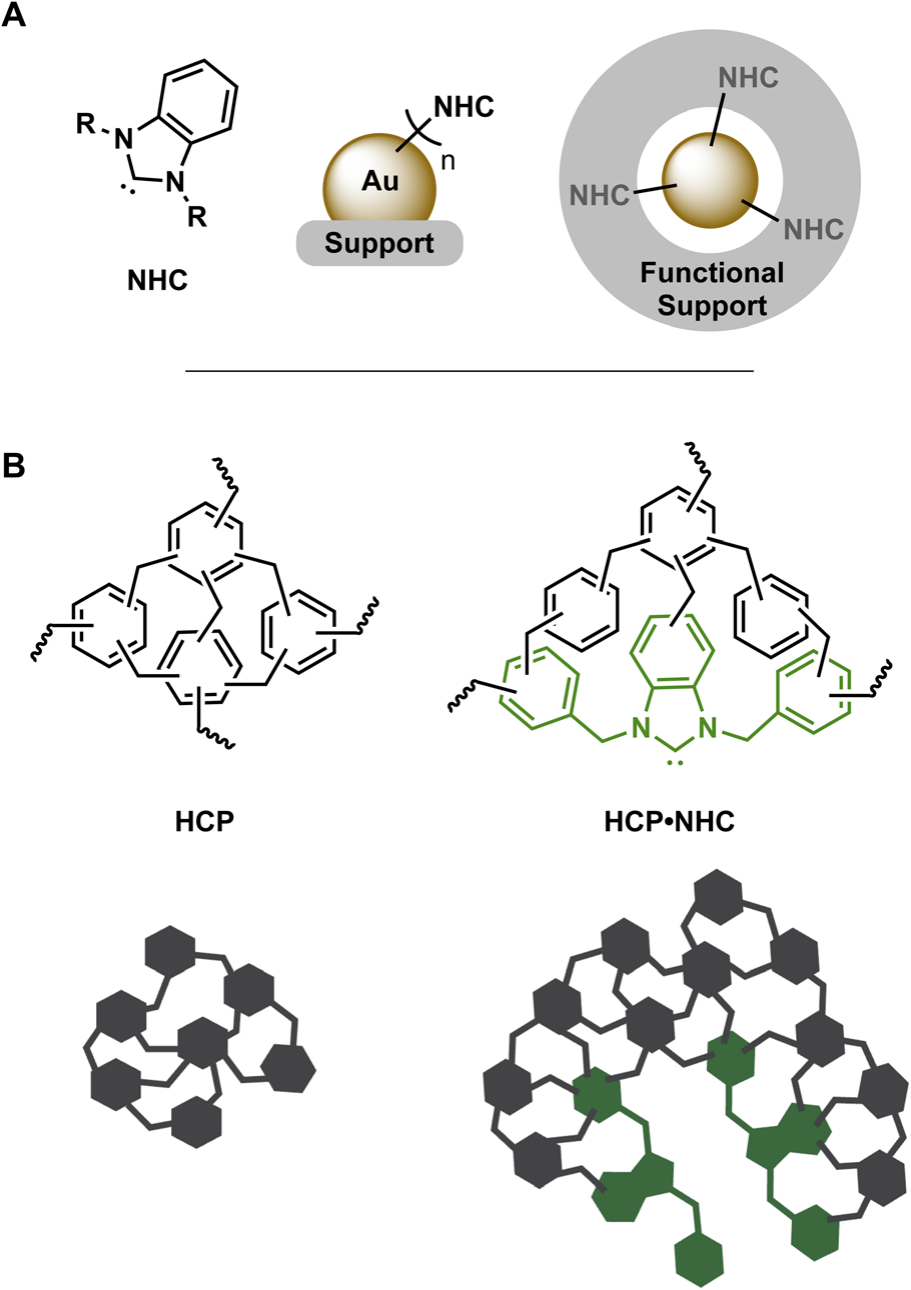
(A) left to right: general structure benzimidazole based NHC, NHC@AuNP on solid support and AuNP supported in NHC containing support structure. (B) Structure and schematic of HCP/HCP·NHC structures.

In the pursuit of highly active and selective heterogenous catalysts, the development of simple yet novel approaches to metal-based nano catalysts supported on functional materials is of huge interest. The successful application of such catalysts in challenging conditions requires an adequate degree of stabilization to mitigate decomposition, aggregation, or sintering, while maintaining good catalytic activity.^1^ In this context, N-heterocyclic carbene stabilized gold nanoparticles (NHC@AuNP; Fig. 1A) recently emerged as promising systems to investigate the interplay of NHCs as modular surface anchors and AuNPs as active nano catalysts.^2^ NHC@AuNPs benefit from the structural versatility of NHC precursors as well as their strong and tunable bond towards metallic NPs and surfaces,^3^ forming well defined and functional protective ligand shells.^4^ In addition, AuNPs contribute with their intrinsic photophysical properties and their nano-sized enhanced catalytic activity.^5^ Despite the high levels of stabilization bestowed by NHCs, NHC@AuNPs often require support structures in order to withstand harsh catalytic conditions or to grant simple catalyst recyclability.^6^ Therefore, the production of “functional supports” by the combination of NHC ligands and support structures is desirable but examples are currently limited.^7^

Hyper-crosslinked polymers (HCPs; Fig. 1B) are a subset of porous organic polymer produced *via* simple and low-cost synthetic procedures.^8^ These procedures permit significant structural and functional diversity in HCPs and allow the introduction of monomers featuring distinct coordination sites including nitrogen rich moieties,^9^ thiols,^10^ phosphines^11^ and precursors of NHCs.^12^ The ability to incorporate various inorganic and/or organic catalytic centers make HCPs ideal candidates as catalyst supports.^13^ Furthermore, catalytically active HCPs benefit heavily from their porosity and swelling capabilities^14^ initially used in storage^14*b*,15^ and separation applications.^16^

The incorporation of NHCs in HCP structures has given rise to various polymer networks containing NHC immobilized molecular metals complexes (HCP·NHC–M). The production of these functional supports bestowed improved thermal stability and potential routes to fine tune catalytically activity. These advancements led so far to several examples of catalytically active HCP·NHC–M, utilizing molecular Pd,^12*a*,17^ Ru,^17*c*^ Ir^17*c*^ and Cu^18^ as active metal centers for a variety of metal catalyzed reactions, *e.g.* C–C couplings, hydrogenations and condensation reactions. HCPs offer the ability to consolidate the valuable properties of NHCs and solid supports to produce ideal and cost-efficient porous support materials for the immobilization of molecular and nanostructured catalytic centers. Through the combination of porous organic polymer networks and NHC properties, a series of beneficial properties can be achieved: (1) reduction of NP leaching from the polymer network by strong immobilization of metallic NPs at NHC sites; (2) protection of NPs against ripening or sintering effects by steric confinement in the porous support^19^ and (3) additional surface activation caused by the σ-donation capabilities of NHCs attached to the NP surface.^2*c*,20^

Despite the aforementioned benefits, only a few examples of porous organic polymers with knitted NHCs as stabilizers of metallic NPs exist.^7*b*,21^ In the case of HCP·NHC stabilized metallic NPs (HCP·NHC@NP) – to the best of our knowledge – no examples are currently present in literature. Therefore, we present here the synthesis and detailed characterization of the first example of HCP·NHC@NP and their evaluation in a catalytic model reaction.

We used the well-established chemistry of NHC@AuNP to synthesize HCP·NHC@AuNPs in a two-step procedure by the immobilization of an Au(i) precursor in an HCP·NHC network followed by the reduction of immobilized NHC–Au(i) complexes to obtain HCP·NHC@AuNPs. The synthesis process was thoroughly monitored to confirm NHC formation, metalation and subsequent immobilization of AuNPs in the HCP network.

## Results and discussion

### Synthesis of HCP·NHC@AuNP

The precursor for HCP·NHC@AuNP, HCP·BIMZ was prepared according to literature procedure with slight iterations.^12*a*^ To form the HCP a one-step polymerization procedure was used were *N*,*N*-diphenylbenzimidazolium chloride (BIMZ), benzene, and dimethoxymethane (DMM) are reacted in the presence of FeCl_3_ catalyst using 1,2-dichloroethane as solvent, yielding HCP·BIMZ. The resulting polymer was characterized in detail by ^13^C solid-state nuclear magnetic resonance spectroscopy (ssNMR), elemental analysis (EA), Brunauer–Emmett–Teller (BET) surface area analysis, powder X-ray diffraction (PXRD) and Fourier-transformed infrared spectroscopy (FT-IR; see ESI† for details). The obtained results are in good agreement with previous reported networks.^12*a*^

**Scheme 1:**
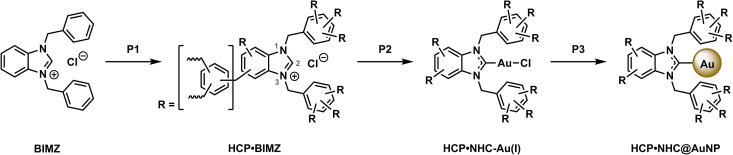
Synthesis of HCP·NHC@AuNP. P1 polymerization: BIMZ, benzene, DMM, FeCl_3_ in 1,2-dichloroethane at 80 °C for 24 h. P2 complexation: KHMDS, toluene, 1 h, RT followed by [Au(DMS)Cl], DCM, 16 h, RT. P3 reduction: NaBH_4_, DCM, 16 h, RT.

To obtain the free carbene compound HCP·NHC, HCP·BIMZ was finely ground, dried *in vacuo* and transferred into an Ar-filled glovebox. HCP·BIMZ was swelled in toluene and potassium bis(trimethylsilyl)amide (KHMDS) was added to deprotonate all carbenic positions (C^2^) throughout the polymer network, yielding HCP·NHC. HCP·NHC was resuspended in DCM and chloro(dimethyl sulfide)gold(i) ([Au(DMS)Cl]) was added affording HCP·NHC–Au(i) (Scheme 1, P2). Subsequently, HCP·NHC–Au(i) was resuspended in DCM and sodium borohydride (NaBH_4_) was added as reducing agent. The resulting suspension was vigorously stirred overnight and worked up under air (P3). No characteristic color change associated with the successful formation of plasmonic AuNPs was observed. The solids were washed with EtOH and DCM to remove excess reducing agent and unbound AuNPs. Subsequent drying of the brown solid yielded the final HCP·NHC@AuNP composite.

### Characterization of AuNP immobilization

The synthesis of HCP·NHC@AuNP was closely monitored, confirming successful complexation and AuNP formation. Analytical data obtained through ^13^CssNMR, FT-IR, PXRD and EA confirmed that the HCP structure did not decompose during the complexation and reduction steps. Unfortunately, all attempts to characterize the NHC–Au bonds (*e.g.* through the ^13^C ssNMR shift) were unsuccessful. Therefore, detailed monitoring by XPS was used to identify the expected NHC–Au coordination.

In order to characterize any changes of the polymer and to confirm the NHC–Au bond formation, XPS analysis was performed on HCP·BIMZ, HCP·NHC–Au(i), and HCP·NHC@AuNP respectively. XPS analysis was performed on sample films, drop casted on silicon wafer substrates. Obtained XPS data of HCP·BIMZ is consistent with previously reported benzimidazolium systems.^22^ Deconvolution of C 1s spectra reveal contributions of C–N (285.2 eV) and aromatic and aliphatic C=C/C–C (284.5 eV), while the N 1s spectra display peaks for N–C (401.4 eV) and N=C (399.2 eV). Organic contributions can be attributed to the aromatic polymer network (C=C) and corresponding crosslinks (C–C) as well as the embedded BIMZ structure with contributions of the benzimidazolium structure (N–C) and the quaternary N (N=C) (see ESI, Fig. S1†).

**Fig. 2:**
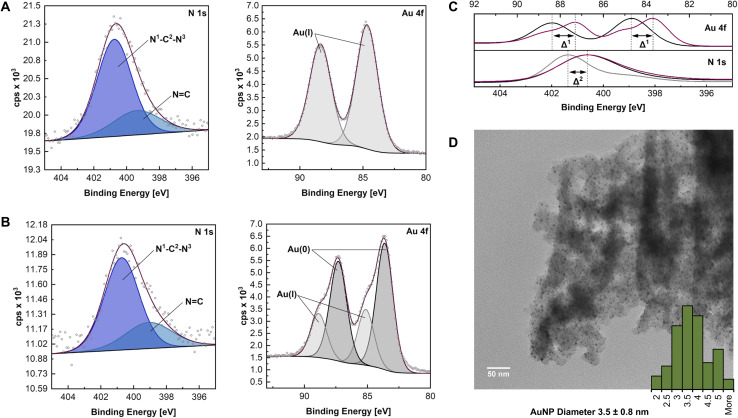
(A and B) High resolution N 1s and Au 4f scans of HCP·NHC–Au(i) and HCP·NHC@AuNP respectively; (C) comparison of N 1s and Au 4f scan and corresponding *Δ*^1/2^*via* envelopes of HCP·BIMZ (gray), HCP·NHC–Au(i) (black) and HCP·NHC@AuNP (red); (D) TEM micrograph of HCP·NHC@AuNP.

Successful preparation of HCP·NHC–Au(i) was confirmed by the presence of Au 4f signals (Au 4f_5/2_ at 88.4 eV and Au 4f_7/2_ at 84.7 eV). Obtained Au 4f binding energies are in accordance with literature values of Au(i) indicating the successful formation of NHC–Au(i) bonds.^22*a*,23^ C 1s scans show identical organic contributions as compared to HCP·BIMZ, while the binding energy of N–C in the N 1s is shifted to 400.7 eV (*Δ*^2^ ∼ 0.7 eV) indicating the formation of the NHC structure (N^1^–C^2^–N^3^; Fig. 2A).^22*a*^

The final material HCP·NHC@AuNP shows characteristic NHC contributions as stabilizers of Au nanomaterials. The peaks for N–C and N=C in the N 1s spectrum occur at binding energies of 400.7 eV and 398.8 eV, respectively, and are typical for benzimidazolium NHCs coordinated to AuNPs.^22^ The signal at 400.7 eV is attributed to the NHC donor moiety N^1^–C^2^–N^3^ bound to the AuNP surface, while the C=N^1/3^ contribution indicates the presence of quaternary N of the BIMZ structure embedded in the polymer network. Interestingly, the Au 4f signal splits into four components attributed to a mix of Au(i)/Au(0) present on the AuNP surface, as previously reported by our group and others.^6*a*,24^ Observed binding energies for Au(i) (Au 4f_5/2_ at 88.9 eV and Au 4f_7/2_ at 85.1 eV) are shifted compared to values obtained by HCP·NHC–Au(i). While the binding energies at 87.3 eV and 83.6 eV are assigned to Au(0) (Fig. 2B and C).

The immobilization of AuNP in the polymer network can be followed *via* BET measurements as the occupation of pore volume by gold should result in an apparent BET surface area and pore volume change. Prior to complexation, HCP·BIMZ has a surface area of 888 m^2^ g^−1^ (for BET parameters see ESI, Table S1†). However, upon complexation (formation of HCP·NHC–Au(i)) the surface area decreased by up to 30%, yielding a surface area of 620 m^2^ g^−1^. The surface area then remained relatively unchanged after reduction to HCP·NHC@AuNP, due to comparable spatial demand of molecular and nanosized Au in the porous structure (see ESI, Fig. S5†). Analysis of the sorption isotherms correspond both to type I and type IVa isotherms with uptake at low relative pressures indicative of microporosity and capillary condensation resulting in hysteresis upon desorption, indicating meso/microporosity of the obtained materials.

In order to visualize the immobilization of AuNPs in the HCP·NHC@AuNP TEM was performed. HCP·BIMZ shows only the organic amorphous structures characteristic for HCPs.^25^HCP·NHC–Au(i) contains a small amount of spherical AuNPs due instability of NHC–Au(i) complexes incorporated in the material under ambient conditions. TEM images of HCP·NHC@AuNP display small spherical AuNPs with average size of ∼3.5 nm and a narrow size distribution (Fig. 2D). Furthermore, all AuNP are immobilized in the HCP without any apparent particle leaching from the polymer network, confirming the strong immobilization achieved by the NHC bond to the AuNPs and successful metalation of the precursor HCP·NHC–Au(i).

### Catalytic decomposition of 4-nitrophenol by HCP·NHC@AuNP

**Fig. 3:**
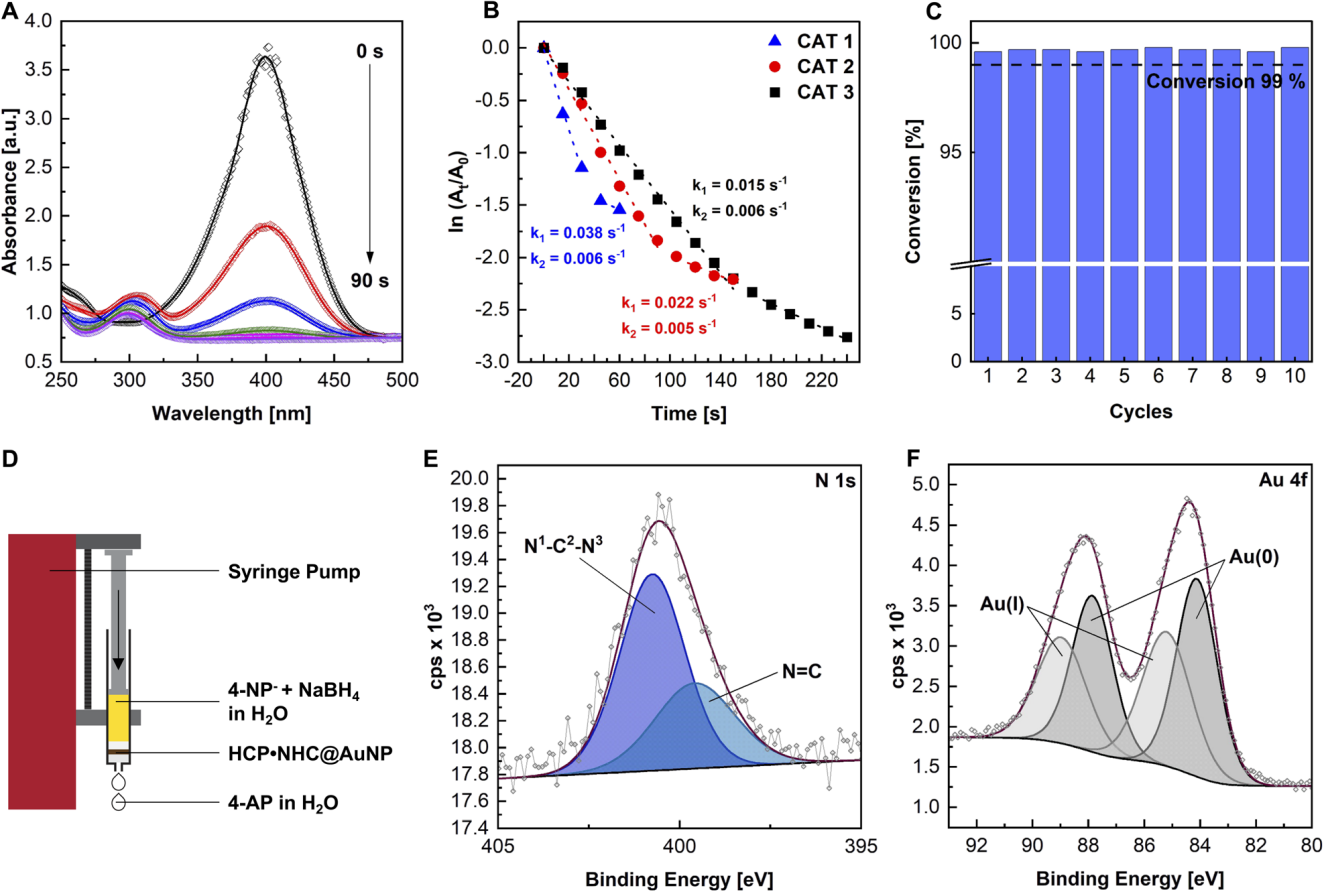
(A) UV-vis absorption spectra of the catalytic reduction of 4-NP to 4-AP at different reaction times using conditions CAT 1; (B) kinetics and corresponding rate constants the reduction reaction of 4-NP^−^ to 4-AP with different HCP·NHC@AuNP loadings (CAT 1–3); (C) conversion of 4-NP^−^ over 10 consecutive cycles in the flow reactor (flow 0.25 mL min^−1^); FCAT 1; (D) schematic of the flow reactor setup; (E) high resolution XPS of N 1s and (F) Au 4f scans after 10 flow-through cycles.

In order to evaluate the catalytic properties of HCP·NHC@AuNP the reduction of 4-nitrophenol (4-NP) was chosen as a model reaction. The reaction follows first-order kinetics according to the classic Langmuir–Hinshelwood mechanism and can be monitored using UV-vis absorption *via* a decrease of the 4-nitrophenolate (4-NP^−^ peak at ∼400 nm (Fig. 3A)). The catalytic experiments were performed using HCP·NHC@AuNP as catalyst, NaBH_4_ as hydride source, H_2_O as solvent and a weight percent ratio between 4-NP/m[Au]/NaBH_4_ of 1 : 3.4 : 421 (see ESI, Table S4†).^10*a*^ The Au content of the presented catalysts HCP·NHC@AuNP was determined by inductively coupled plasma mass spectrometry (ICP-MS) to be 7.27 wt%, which indicates a significant higher loading (∼50%) compared to previously reported HCP materials loaded with AuNPs which did not contain any NHC binding sides (see ESI, Fig. S8†).^10*a*^

The reaction catalyzed by HCP·NHC@AuNP using reaction conditions CAT 1 (see ESI, Table S4†), proceeds with an initial rate constant (*k*_1_) of 0.038 s^−1^ and continues with a second slower rate constant (*k*_2_) of 0.006 s^−1^. *k*_2_ is attributed to the loss of available active Au surface due to the slow detachment of the product 4-aminophenol (4-AP). Adjusting the used Au loading by lowering the Au contribution to 1 : 1.7 : 421 (CAT 2) and 1 : 0.9 : 421 (CAT 3) respectively, resulted in a linear correlation between Au loading and the observed rate constants *k*_1_ = 0.022 s^−1^ (CAT 2) and *k*_1_ = 0.015 s^−1^ (CAT 3), respectively (Fig. 3B). It is worth noting, that no reaction was observed in the absence of HCP·NHC@AuNP nor did the polymer network (HCP·BIMZ) display any catalytic activity.

Observed rate constants display exceptionally high activity of HCP·NHC@AuNP even when compared to highly active dispersed NHC@AuNPs and polymer supported AuNPs (see ESI for comparison, Table S6†). The high activity can be attributed to the high Au loading compared to other AuNP systems but is nevertheless surprising given the hydrophobic character of HCP·NHC@AuNP used in aqueous conditions. In order to accurately monitor the reaction, the reaction mixture was diluted by 2-fold keeping the wt% ratios of reagent and catalyst identical. Dilution resulted in an up to 8-fold slower reaction time allowing a more precise UV-vis monitoring of the catalytic conversion (see ESI, Fig. S14†).

Given the high catalytic activity the catalytic recyclability of HCP·NHC@AuNP was investigated in a “flow-reactor” setup. As “flow-reactor” a dense pad of HCP·NHC@AuNP in a syringe was used (Fig. 3D; for conditions ESI Table S5†) utilizing a syringe pump to push the reaction solution thought the catalyst pad. The recycling experiments were performed under constant flow and revealed the reusability of HCP·NHC@AuNP for up to 10 cycles without any significant loss of activity when reaction conditions FCAT 1 are used (Fig. 3C). Further ICP-MS analysis was used to determine the amount of leached Au. Interestingly, after 10 cycles a gold content of 7.25 wt% was found, which is almost identical to the starting material which contained 7.27 wt%, demonstrating that within the error of the measurements no Au was leached from HCP·NHC@AuNP. Corresponding XPS data of recycled HCP·NHC@AuNP show the full retention of the material composition (Fig. 3E). However, TEM micrographs of recycled HCP·NHC@AuNP show some ripening of AuNPs which seem not to have a significant influence on the catalytic performance (see ESI, Fig. S21†).

With the HCP·NHC@AuNP flow-reactor successfully setup, its performance under different reaction conditions were evaluated. With the current experimental setup, a maximum flow rate of 0.5 mL min^−1^ (FCAT 5) was achieved while maintaining conversion rates of >99% over 10 consecutive cycles. Further, the catalyst pad size was investigated showing the limits of the used setup based on the total mass and consistency of used HCP·NHC@AuNP. Reducing the catalyst loading to a mass of below 0.006 g (FCAT 2) gave only partial conversion due to the formation of an inhomogeneous catalyst pad, which no longer covered the full diameter of the syringe. Nonetheless, HCP·NHC@AuNP shows great potential as solid phase catalyst benefiting from the reactivity of NHC@AuNPs and the porosity of the HCP network.

## Conclusion

Herein, we reported a facile two-step synthesis for the immobilization of AuNPs in a functional HCP support containing NHCs as coordination sites. The hyper-crosslinked polymers containing benzimidazolium as NHC precursors to stabilize AuNPs can be synthesized in excellent yields. Subsequent activation of the HCP with KHMDS followed by the addition of the gold starting material yielded HCP·NHC–Au(i). The final AuNPs containing HCP can be obtained after reduction of HCP·NHC–Au(i). Detailed spectroscopy analysis of HCP·NHC@AuNP proved that the NHCs in HCP indeed stabilize the *in situ* synthesized AuNPs and that NPs are evenly distributed through the HCP solid support. The AuNPs stabilized in the functional HCP support HCP·NHC@AuNP showed high activity in the catalytic reduction of 4-nitrophenol and allowed the repeated application in a flow reactor like system with a conversion of greater than 99% and without leaching of Au from the HCP·NHC network.

This work demonstrates the potential of HCP·NHCs as a solid support to immobilize metallic NPs and its promise in heterogenous catalysis. Further, this concept can be easily translated to other metallic nanoparticles, adding significant versatility to the approached presented.

## Experimental

All reactions were performed using standard Schlenk techniques or an argon-filled glovebox using dry solvents and flame-dried glassware, unless stated otherwise.

The monomer BIMZ,^26^HCP·BIMZ (see ESI† for experimental details)^12*a*^ and [Au(DMS)Cl]^27^ were synthesized according to literature procedures.

### Synthesis of HCP incorporated NHC–Au(i) complexes (HCP·NHC–Au(i))‡‡HCP·NHC–Au(i) is unstable under ambient conditions, rapidly triggering uncontrolled formation of polydisperse AuNPs within 16 h. Decomposed HCP·NHC–Au(i) show comparable XPS contributions as observed in HCP·NHC@AuNP (see ESI, XPS Fig. S3; TEM Fig. S7B†).

HCP·BIMZ (0.100 g containing 0.042 mmol carbenic positions) was pre-soaked in toluene (2 mL, for 10 min) before solid KHMDS (0.009 g, 0.046 mmol, 1.1 eq.) was added in one portion. The resulting suspensions was stirred for 1 h at RT and subsequently washed with toluene (1 × 20 mL) and DCM (2 × 20 mL). The resulting solid was resuspended in DCM (2 mL) and DMS–Au–Cl (0.012 g, 0.043 mmol, 1.1 eq.) was added and stirred for 16 h at RT under exclusion of light. The final suspension was washed with DCM (3 × 20 mL) and subsequent drying *in vacuo* yielded in HCP·NHC–Au(i).

### Preparation of HCP·NHC immobilized AuNPs (HCP·NHC@AuNP)

Previously obtained HCP·NHC–Au(i) was suspended in DCM (2 mL) and NaBH_4_ (0.006 g, 0.147 mmol, 3.5 eq. based on initial used HCP·BIMZ) was added. The resulting mixture was vigorously stirred for 16 h. Under ambient conditions, the solid was washed with EtOH (tech., 1 × 10 mL) and DCM (2 × 10 mL). The washed solid was dried *in vacuo* yielding in HCP·NHC@AuNP as brown powder.

### Catalytic decomposition of 4-nitrophenol (4-NP)

The catalytic experiments were conducted according to literature procedures in order to grant comparability (see ESI for conditions, Table S4†).^10*a*^

All experiments were carried out under ambient conditions and monitored *in situ* by a UV-vis spectrometer. All reactions were conducted using open quartz cuvettes, in a total reaction volume of 2 mL and under constant stirring (600 rpm) at RT.

Typically, a fresh solution of NaBH_4_ in H_2_O (16 mg mL^−1^, 1 mL) was mixed with a aq. solution of 4-NP (0.038 mg mL^−1^, 1 mL) followed by the solid catalyst HCP·NHC@AuNP giving the final reaction mixture. The reaction progress was monitored *in situ* by UV-vis measurements at intervals of 15/30 s until no absorbance change at 400 nm was visible.

### Catalytic decomposition of 4-NP in an improvised flow reactor

Under ambient conditions, a 1 mL syringe with cotton-plug was charged with a pad of HCP·NHC@AuNP and covered with an additional pad of cotton. The reactive filter pad was primed with H_2_O (1 × 0.5 mL), subsequently a mixture of 4-NP (0.019 mg mL^− 1^, 0.5 mL) and NaBH_4_ (8 mg mL^− 1^, 0.5 mL) was added to the syringe. The syringe was mounted in a syringe pump and the reaction mixture was passed through the pad with differing flow rates (see ESI, Table S5†).

Recycling was performed by washing the HCP·NHC@AuNP containing filter pad by two portions of H_2_O (1 mL) and subsequent priming with H_2_O as described above. Product identification was performed *via* comparison of UV-vis maxima (see ESI, Fig. S12†).

## Author contributions

M. R. R. and R. W. designed the study. C. E., E. S. and L. G. performed the experiments. C. E. and M. R. R. analyzed the XPS data. M. R. R., J. C. and R. W. supervised the study. All authors discussed the results. The manuscript was written through contributions of all authors.

## Conflicts of interest

There are no conflicts to declare.

## Supplementary Material

NA-005-D2NA00799A-s001
