# Supplementary material for: Hyper crosslinked polymer supported NHC stabilized gold nanoparticles with excellent catalytic performance in flow processes
Source: Nanoscale Adv. 2022 Nov 21;5(4):1095–101. doi: 10.1039/d2na00799a (PMC9926895; doi:10.1039/d2na00799a)
Supplement: NA-005-D2NA00799A-s001 [file NA-005-D2NA00799A-s001.pdf]

Electronic Supplementary Material (ESI) for Nanoscale Advances.
This journal is © The Royal Society of Chemistry 2022

Electronic Supporting Information

HCP Supported NHC Stabilized Gold Nanoparticles with Excellent Catalytic Performance in Flow Processes

Constantin Eisen[a], Lingcong Ge[a], Elena Santini[b], Jia Min Chin[c], Robert T. Woodward[b*], Michael R. Reithofer[a*]*

[a] Institute of Inorganic Chemistry, Faculty of Chemistry, University of Vienna, Währinger Straße 42, 1090 Vienna, Austria.
email: michael.reithofer@univie.ac.at

[b] Institute of Material Chemistry and Research, Faculty of Chemistry, University of Vienna, Währinger Straße 42, 1090 Vienna, Austria.
email: robert.woodward@univie.ac.at

[c] Institute of Inorganic Chemistry – Functional Materials, University of Vienna, Währinger Straße 42, 1090 Vienna, Austria.
email: jiamin.chin@univie.ac.at

Materials and Methods **2**
Synthesis of HCP•BIMZ **3**
XPS Spectra **4**
High Resolution XPS Spectra of HCP•BIMZ 4
High Resolution XPS Spectra of HCP•NHC-Au(I) 4
High Resolution XPS Spectra of HCP•NHC@AuNP 5
N_2 Sorption Isotherms **6**
Textural Properties of HCP•BIMZ, HCP•NHC-Au(I) and HCP•NHC@AuNP **7**
TEM Micrographs **8**
Elemental Analysis of HCP•BIMZ, HCP•NHC-Au(I) and HCP•NHC@AuNP **9**
TGA of HCP•BIMZ, HCP•NHC-Au(I) and HCP•NHC@AuNP **10**
PXRD of HCP•BIMZ, HCP•NHC-Au(I) and HCP•NHC@AuNP **10**
^{13}C (CP/MAS) ssNMR Spectra of HCP•BIMZ, HCP•NHC-Au(I) and HCP•NHC@AuNP **11**
FT-IR of HCP•BIMZ, HCP•NHC-Au(I) and HCP•NHC@AuNP **11**
Catalytic Reduction of 4-NP **12**
Comparison of Different Reaction Conditions Used in Direct Reaction Monitoring 12
UV-Vis Absorption Spectra of the Catalytic Reduction of 4-NP^- to 4-AP 13
XPS Spectra and TEM Micrograph of Recovered HCP•NHC@AuNP 14
Flow Catalysis 15
XPS Spectra and TEM Micrograph of Recovered HCP•NHC@AuNP from Flow Catalysis 16
Comparison of Different Polymer or NHC Stabilized Gold Catalyst for Catalytic Reduction of 4-NP^- 17
References **18**

Materials and Methods

Commercially available reagents were used without further purification. Dry solvents were obtained from Acros/Fischer or Sigma Aldrich and stored over activated molecular sieves (3 Å). Ultra-pure water (18.2 MΩ.cm) was obtained by a Millipore Milli-q Advantage water purification system.

X-ray photoelectron spectroscopy (XPS) was performed on a Nexsa Photoelectron Spectrometer (Thermo Fisher Scientific, UK). Samples were drop casted as suspensions in DCM onto silicon wafer to form thin films for analysis. Element specific high-resolution spectra for Carbon (C 1s 279-298 eV) Nitrogen (N 1s 392-410 eV) and Gold (Au 4f 80-96 eV) with step sizes of 0.1 eV and a pass energy of 50 eV were acquired. All measurements were performed using Al-Kα X-rays with a spot size of 400 µm. Obtained spectra were evaluated using the Advantage software package (v5.9929/build 06752) provided by Thermo Fisher Scientific.

Solid-state NMR (ssNMR) was carried out on a Bruker Avance NEO 500 wide bore system (Bruker BioSpin, Rheinstetten, Germany) using a 4 mm triple resonance magic angle spinning probe. Between 15 - 25 mg of material was packed into a 4 mm zirconia CRAMPS rotor. The resonance frequency for ^{13}C NMR was 125.78 MHz, the MAS rotor spinning was set to 14 kHz. Cross polarisation was achieved by a ramped contact pulse with a contact time of 3 ms. During acquisition ^{1}H was high power decoupled using SPINAL with 64 phase permutations. The ^{1}H π/2 pulse was 2.5 µs, the relaxation delay was set to 4 s, and with roughly 2000 scans a sufficient signal to noise was achieved. The chemical shifts for 13C are reported in ppm and are referenced external to adamantane by setting the low field signal to 38.48 ppm.

UV-Vis measurements were conducted with an Agilent Technologies G1103A using open quartz cuvettes. Stirring was provided by an external magnetic stirrer.

Fourier-transform infrared spectroscopy (FT-IT) was performed on finely ground samples in the range 350-4000 cm^{-1} using a Tensor II FT-IR Spectrometer from Bruker.

Thermal analyses (TGA) were performed using a Discovery TGA (TA instruments, USA). Approximately 10 mg of each sample was heated from room temperature under air flow (100 $mL\cdot min^{-1}$) to 110 °C at a rate of 10 $°C\cdot min^{-1}$ and held at this temperature for 15 min. Following the isothermal step, samples were heated to 800 °C at a rate of 10 $°C\cdot min^{-1}$.

Elemental analysis (EA) was performed using a Eurovector EA 3000 CHNS-O Elemental Analyser. Between 0.75 and 3.0 mg of each sample was weighed into tin vials (4×6 mm) for each individual run and each sample was ran at least in duplicate. Sample weighing is done using a micro balance (Sartorius, ME 5 OCE) for accuracy. The operating temperatures for the combustion and reduction were 1000 °C (1480 °C for O analysis) and 750 °C, respectively, with high purity helium (99.999+) used as a carrier gas.

Total Au-loading was determined using *inductively coupled plasma mass spectrometry* (ICP-MS) (Agilent 8800 ICP-MS, Vienna, Austria). Samples (~2 mg) were digested in HCl:HNO3 (1:1 v:v) for 48 h in air, followed by heating under reflux for 2 h.

Porous properties were determined using N_2 sorption-desorption isotherms measured at -196 °C using a TriStar II from Micromeritics Instrument Corporation, controlled by the software TriStar II 3020 version 3.02. Samples were degassed for at least 4 h at 120 °C under N_2 using a FlowPrep 060 from Micromeritics Instrument Corporation. Surface areas were calculated using the Brunauer-Emmett-Teller (BET) method on the adsorption branch between 0.05-0.2 P/P_0. Total pore volumes were calculated from the volume of N_2 adsorbed at $P/P_0 = 0.97$ and micropore volumes were determined using the t-plot method for the relative pressure range between 0.15-0.4.

Powder X-ray diffraction (PXRD) measurements were performed on a Malvern Panalytical Empyrean instrument in reflectance mode configuration using Cu $K\alpha_{1+2}$ radiation (wavelength of 1.5406 Å). Measurements were taken using a step size of 0.0263 °2θ, generator settings of 40 mA and 45 kV at a measurement temperature of 25 °C. The scan range was 5 to 50° with a scan step time of 59.4 s.

Transmission electron microscopy (TEM) was measured at the Electron Microscopy Facility at IST Austria using a Phillips Tecnai 12 (120kV) TEM equipped with a CMOS TVIPS TemCam-F216 camera. Prior to measurement, samples suspended in toluene were drop casted onto formvar carbon film on 200 mesh copper grids. TEM micrographs were analyzed using on Image J.

Synthesis of HCP•BIMZ

HCP•BIMZ was synthesized following a procedure reported previously.[1] Briefly, BIMZ (0.670 g, 2 mmol), benzene (0.468 g, 6 mmol), and dimethoxymethane (DMM) (1.360 g, 18 mmol) were added to 1,2-dichloroethane (8 mL). $FeCl_3$ (2.920 g, 18 mmol) was quickly added, and the reaction vessel fitted with a reflux condenser. The reaction was then heated to 40 °C for 5 h to form a pre-network, before being heated at 80 °C overnight to drive the reaction to completion. The resulting polymer was then washed with methanol for 24 h *via* Soxhlet extraction before drying at 60 °C *in vacuo*, yielding **HCP-BIMZ** in quantitative yields.

XPS Spectra

All displayed spectra were deconvoluted using the Avantage software package (v5.9929/build 06752) provided by Thermo Fisher. Displayed baselines were generated by using the smart baseline feature of the software package. Raw data is displayed as gray symbol, generated envelope as red line and peak fits as colored areas.

High Resolution XPS Spectra of HCP•BIMZ

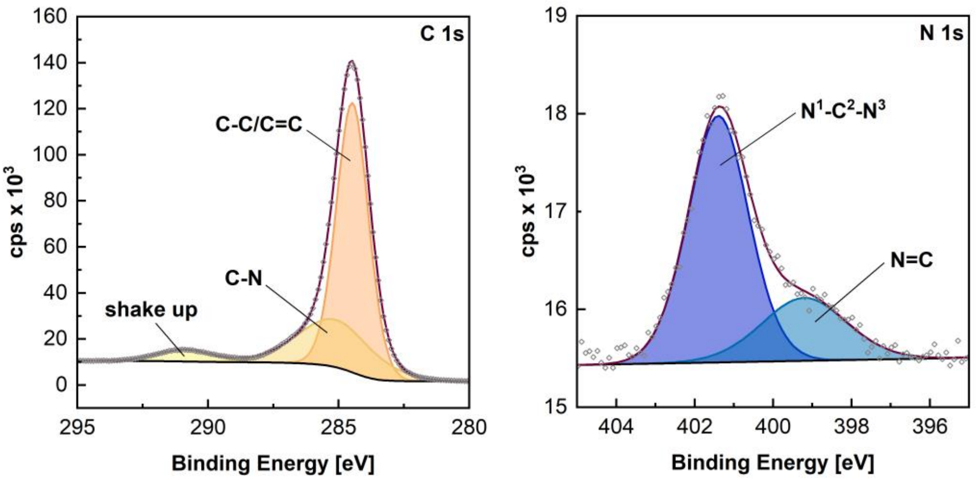

Figure S1. C 1s and N 1s scan of **HCP•BIMZ**.

High Resolution XPS Spectra of HCP•NHC-Au(I)

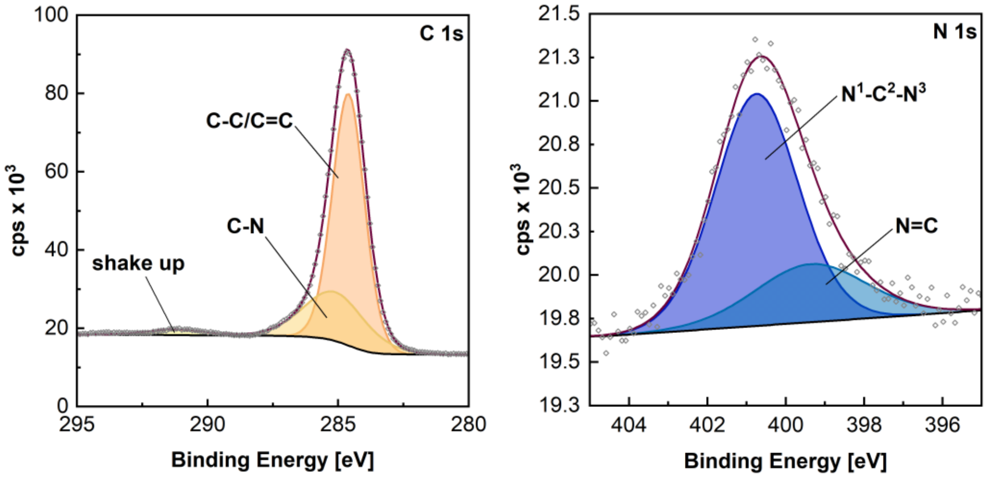

Figure S2. C1 s and N 1s scan of freshly prepared **HCP•NHC-Au(I)**.

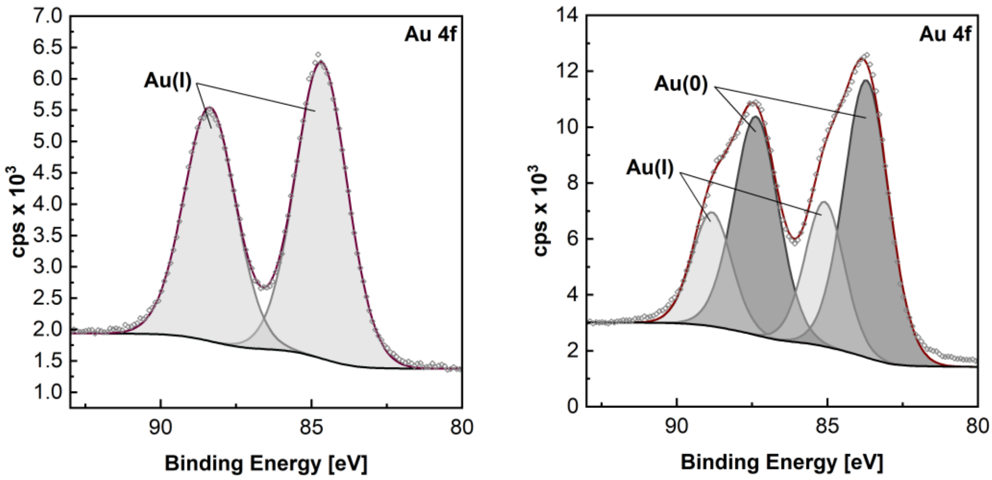

Figure S3. Au 4f scan of freshly prepared (left) and decomposed (right) **HCP•NHC-Au(I)**.

High Resolution XPS Spectra of HCP•NHC@AuNP

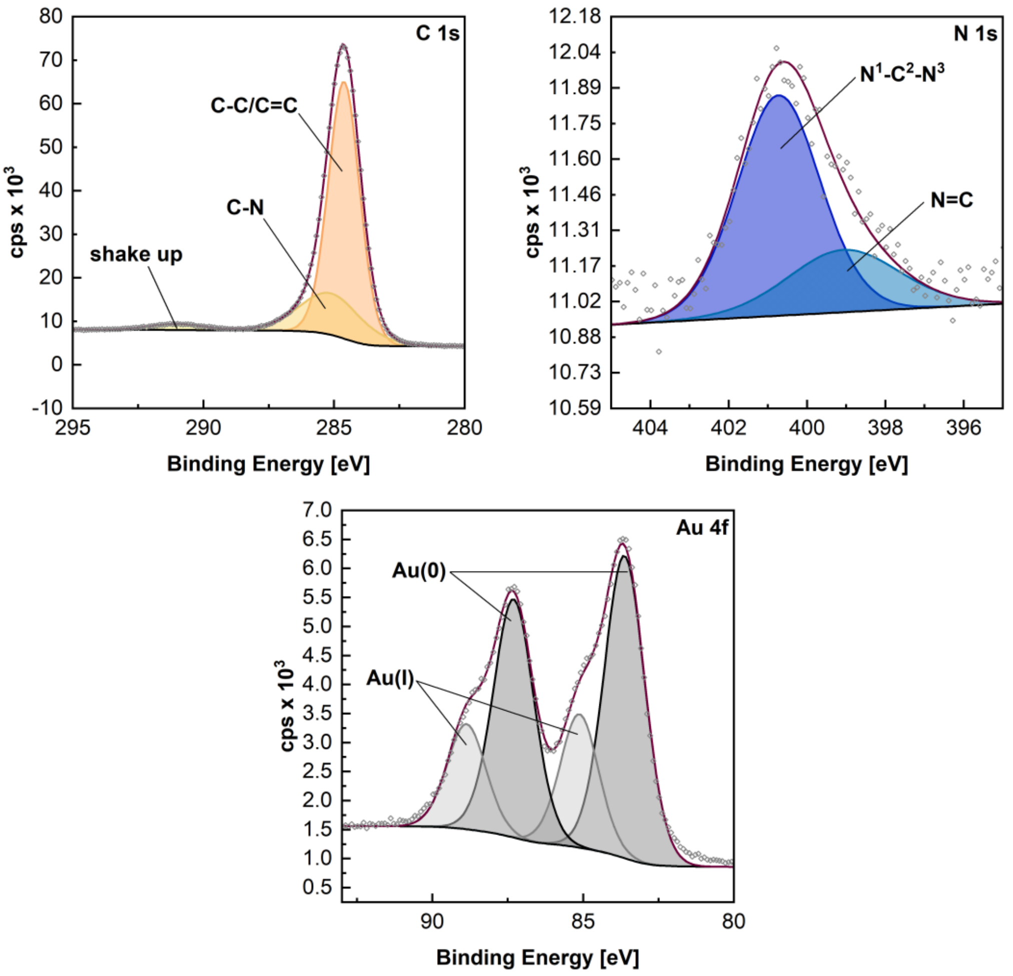

Figure S4. C 1s, N 1s and Au 4f scans of **HCP•NHC@AuNP**.

N_2 Sorption Isotherms

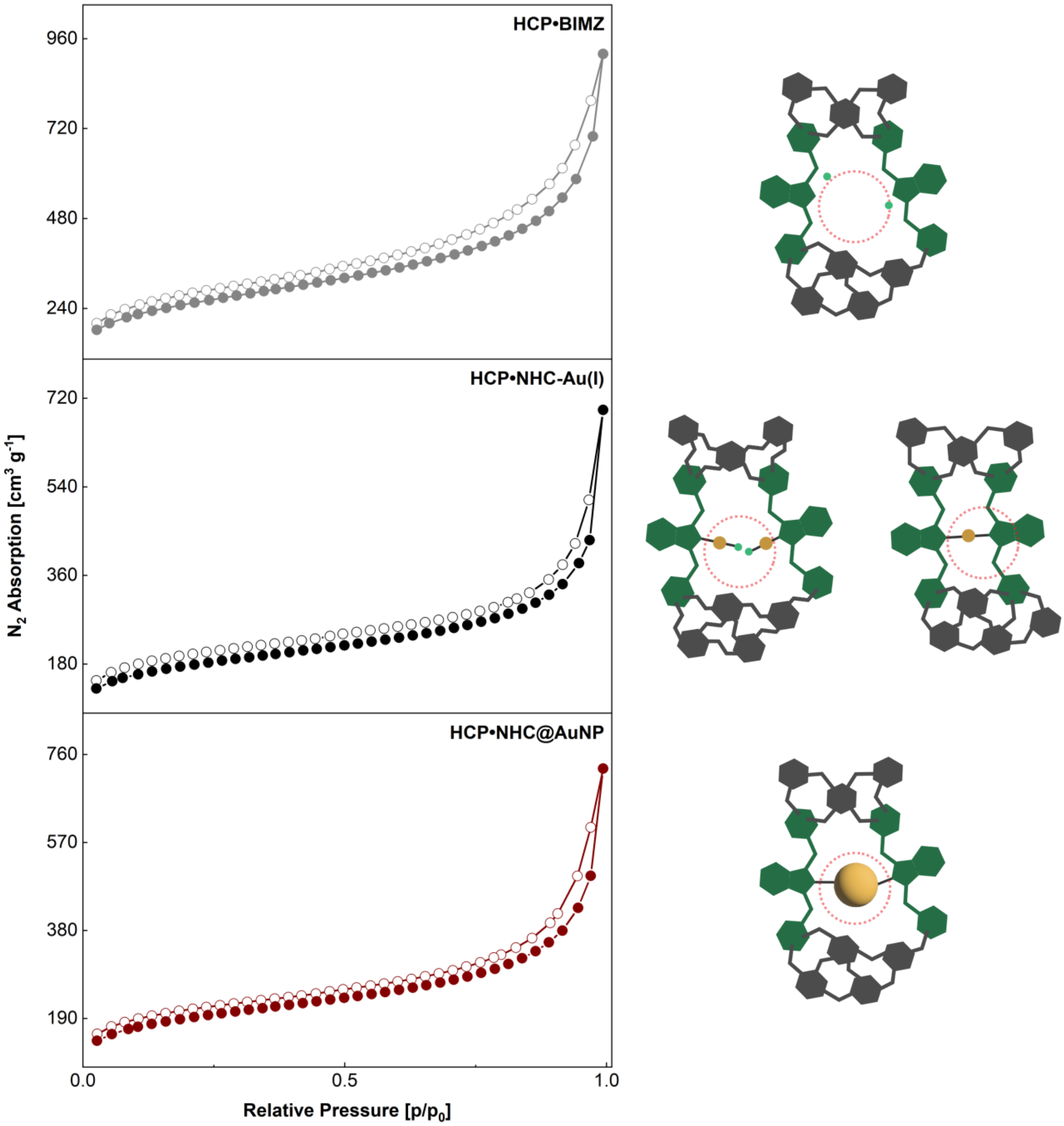

Figure S5. Left: N_2 Adsorption (solid symbols) and desorption (open symbols); right: schematic representation of metalation (green dot = chlorine, yellow dot = Au(I) and yellow sphere = AuNP) influence on available pore volume (red circle).

Textural Properties of HCP•BIMZ, HCP•NHC-Au(I) and HCP•NHC@AuNP

Table S1. Textural Properties of obtained materials.

Material	SA_{BET} [m^2 g^{-1}][a]	V_{MICRO} [cm^3 g^{-1}][b]	V_{TOT} [cm^3 g^{-1}][c]
**HCP•BIMZ**	888	0.17	1.08
**HCP•NHC-Au(I)**	620	0.13	0.67
**HCP•NHC@AuNP**	669	0.15	0.77

[a] Calculated from N_2 sorption isotherms measured at 77 K. [b] Calculated using the t-plot method from N_2 sorption isotherms measured at 77 K. [c] Calculated from N_2 uptake measured at P/P_0 = 0.97.

TEM Micrographs

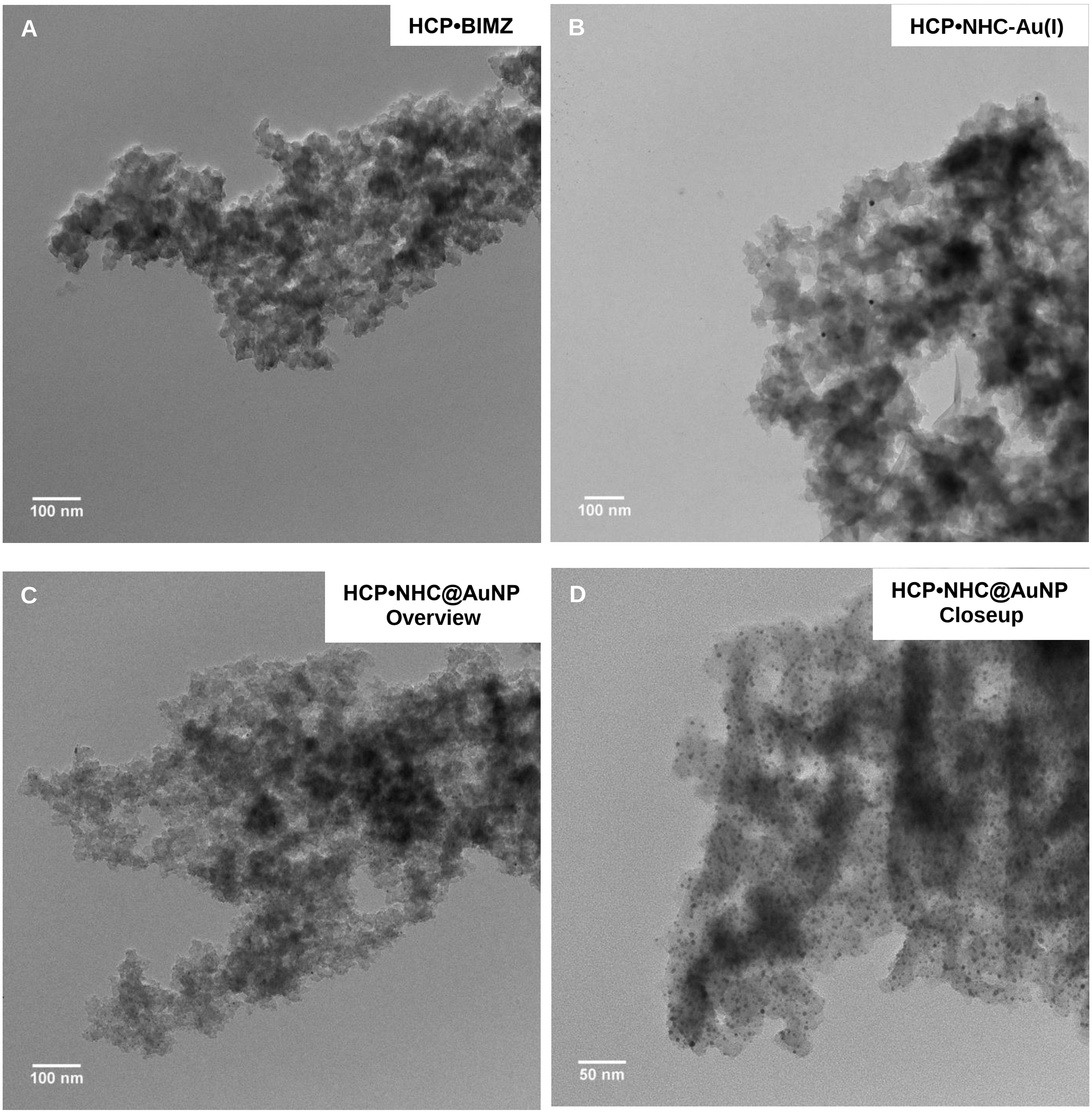

Figure S6. TEM micrographs of A) **HCP•BIMZ**, B) **HCP•NHC-Au(I)** and C-D) **HCP•NHC@AuNP.**

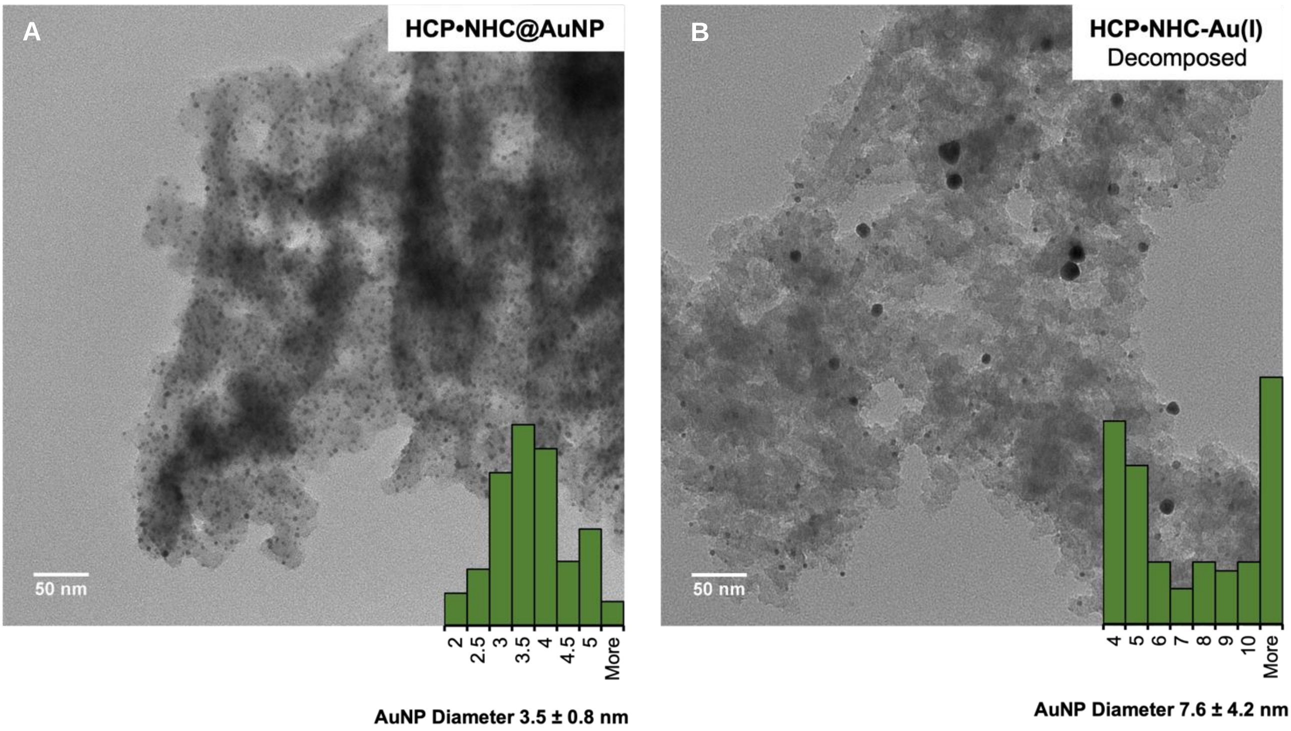

Figure S7. TEM micrographs and statistical evaluation of size distribution of A) decomposed **HCP•NHC-Au(I)** and B) **HCP•NHC@AuNP**. Statistical data based on n = 100 AuNPs.

Elemental Analysis of HCP•BIMZ, HCP•NHC-Au(I) and HCP•NHC@AuNP

Table S2. Overview on EA data of obtained materials.

Material	C [%]	H [%]	N [%]
**HCP•BIMZ**	84.15	5.42	1.16
**HCP•NHC-Au(I)**	67.69	4.23	1.13
**HCP•NHC@AuNP**	73.83	4.60	1.16

TGA of HCP•BIMZ, HCP•NHC-Au(I) and HCP•NHC@AuNP

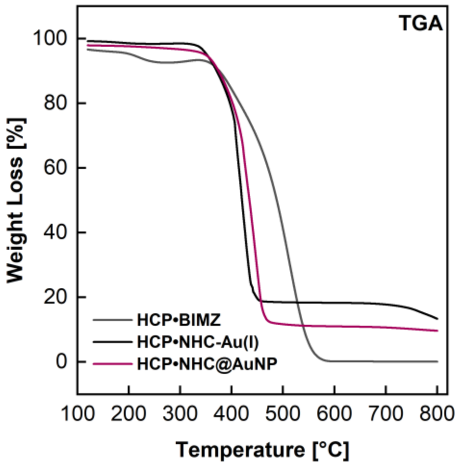

Figure S8. Comparison of TGA curves obtained under air (100 mL·min^{-1} flow rate).

Table S3 Summary of measured and theoretical weight loss.

Material	$Weight_{Start}$ [%][a]	$Weight_{Final}$ [%][b]	ΔWeight [%]	Theo. Au Load [wt%]
**HCP•BIMZ**	97	0	97	/
**HCP•NHC-Au(I)**	99	18	81	9
**HCP•NHC@AuNP**	97	11	88	7

Ramp 10 °C/min, 100-800 °C. [a] Start value 120 °C to ensure removal of all volatiles. [b] End value at 800 °C. [c] Weight difference calculated between 120-800 °C.

PXRD of HCP•BIMZ, HCP•NHC-Au(I) and HCP•NHC@AuNP

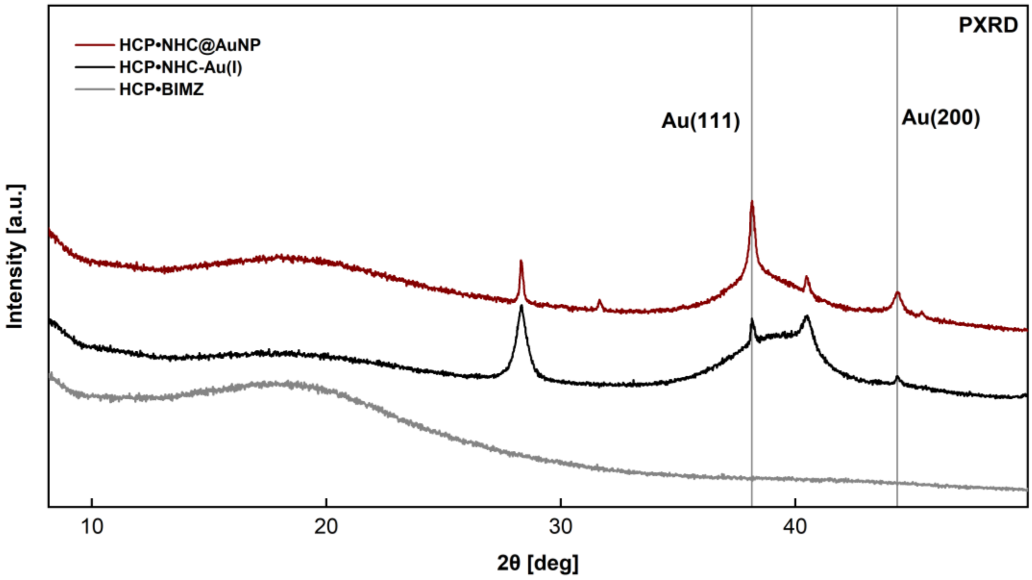

Figure S9. PXRD of obtained materials.

^{13}C (CP/MAS) ssNMR Spectra of HCP•BIMZ, HCP•NHC-Au(I) and HCP•NHC@AuNP

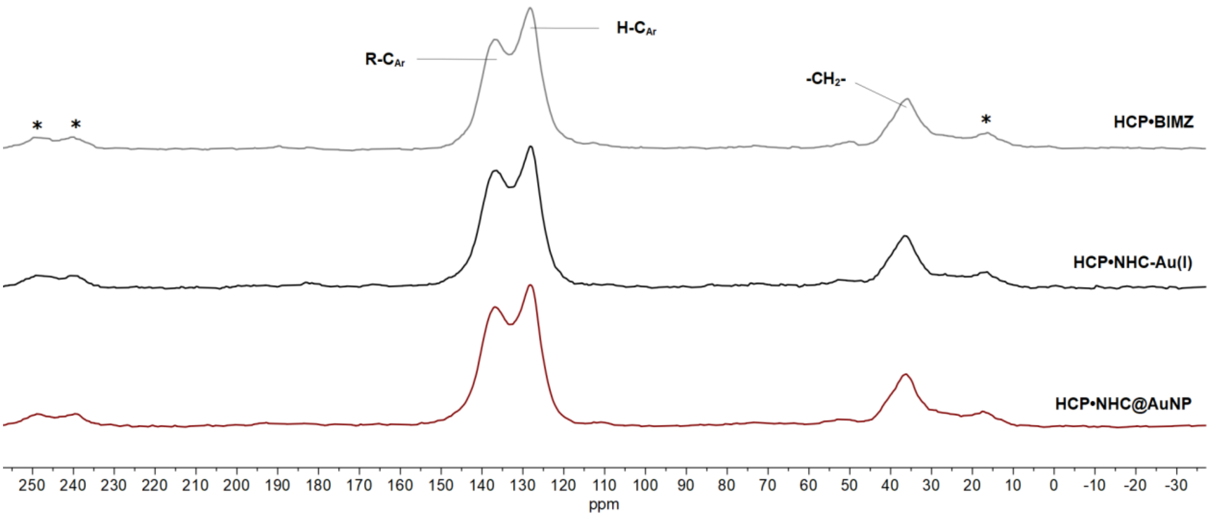

Figure S10. 13C (CP/MAS) ssNMR of obtained materials.

^{13}C ssNMR allows the identification of characteristic peaks of HCP including aromatic R-C$_{Ar}$, H-C$_{Ar}$ and crosslinking -CH$_2$- moiety all associated to the HCP structure.[1-2] The C^2 position of the BIMZ and NHC structure (expected between~140 ppm and ~180 ppm, respectively) could not be detected due comparably low concentration in the HCP network. * are attributed to spinning sidebands.

FT-IR of HCP•BIMZ, HCP•NHC-Au(I) and HCP•NHC@AuNP

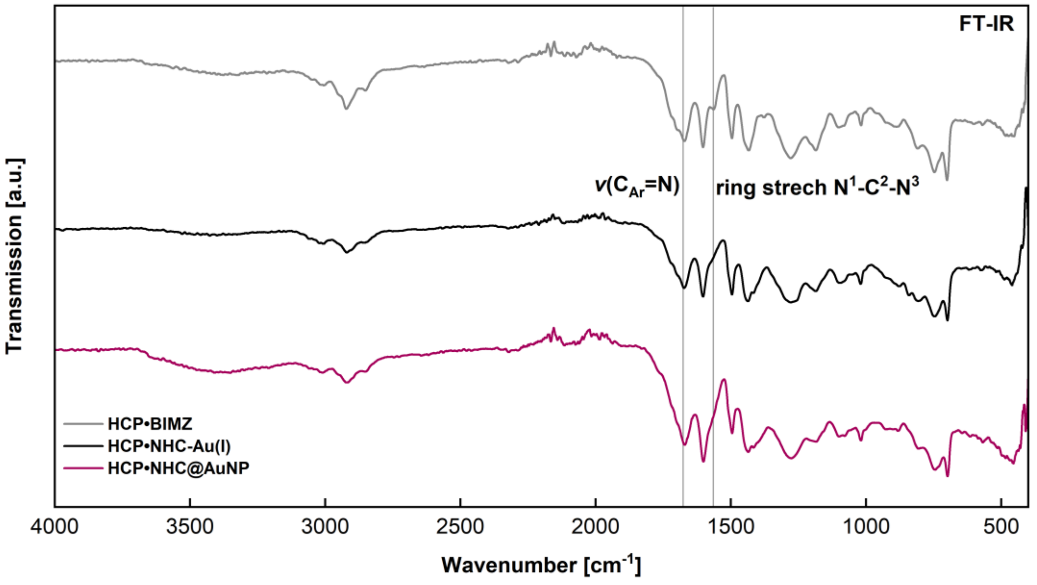

Figure S11. FT-IR spectra of obtained materials.

FT-IR shows characteristic signals for HCPs. The contribution of BIMZ and the corresponding NHC structure is observable by the peaks between 1720-1600 cm^{-1} (ν(C=C)/ν(C=N)) and 1564 cm^{-1} (ring stretch (N^1-C^2-N^3))[3]. Remaining bands in the regions 3000-2900 cm^{-1} (ν(C-H)), 1600-1450 cm^{-1} (ν(C$_{Ar}$=C$_{Ar}$)), and 900-650 cm^{-1} (π(C$_{Ar}$=C$_{Ar}$-H)) are characteristic for the aromatic network of HCPs.[1-2]

Catalytic Reduction of 4-NP

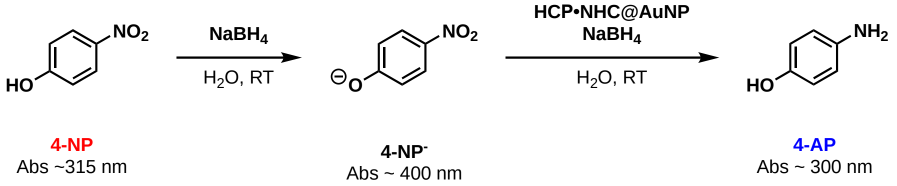

Scheme S1. Reaction scheme of catalytic reduction of 4-NP over 4-NP$^-$ to 4-AP as product.

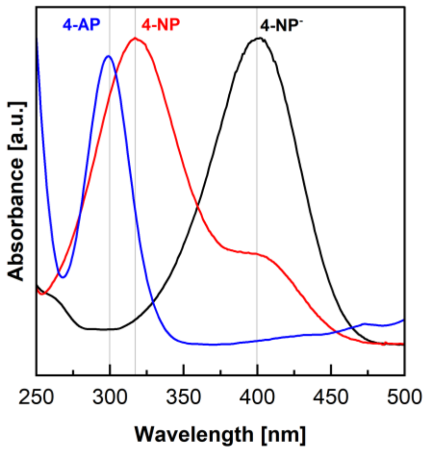

Figure S12. Normalized UV-Vis spectra of 4-NP, 4-NP$^-$ and 4-AP in H_2O.

Comparison of Different Reaction Conditions Used in Direct Reaction Monitoring

Table S4. Conditions for the catalytic reduction of 4-NP.

Catalyst	wt% Ratio [4-NP/m[Au]^a/NaBH$_4$]	Reaction time [s]^b	4-NP$_{Start}$ [mg]	Reaction volume [mL]	HCP•NHC@AuNP Load [mg]	Identifier
**HCP•NHC@AuNP**	1:3.4:421	90	0.038	2	1.8	**CAT 1**
**HCP•NHC@AuNP**	1:1.7:421	150	0.038	2	0.9	**CAT 2**
**HCP•NHC@AuNP**	1:0.9:421	240	0.038	2	0.45	**CAT 3**
**HCP•BIMZ**	/	No reaction	0.038	2	1.8	**CAT 4**
No catalyst	/	No reaction	0.038	2	/	**CAT 5**
**HCP•NHC@AuNP**	1:3.4:421	540	0.019	2	0.9	**CAT 6**
**HCP•NHC@AuNP**	1:1.7:421	1200	0.019	2	0.45	**CAT 7**

HCP•NHC-Au(I) was not tested due to the necessary reductive conditions leading to the uncontrolled formation of **HCP•NHC@AuNP** during the catalytic reaction. ^{a} Au content (wt%) of HCP$_{cat}$ based on ICP-MS results. ^{b} Reaction time until no UV-Vis absorbance change at ~400 nm was observed.

UV-Vis Absorption Spectra of the Catalytic Reduction of 4-NP^{-} to 4-AP

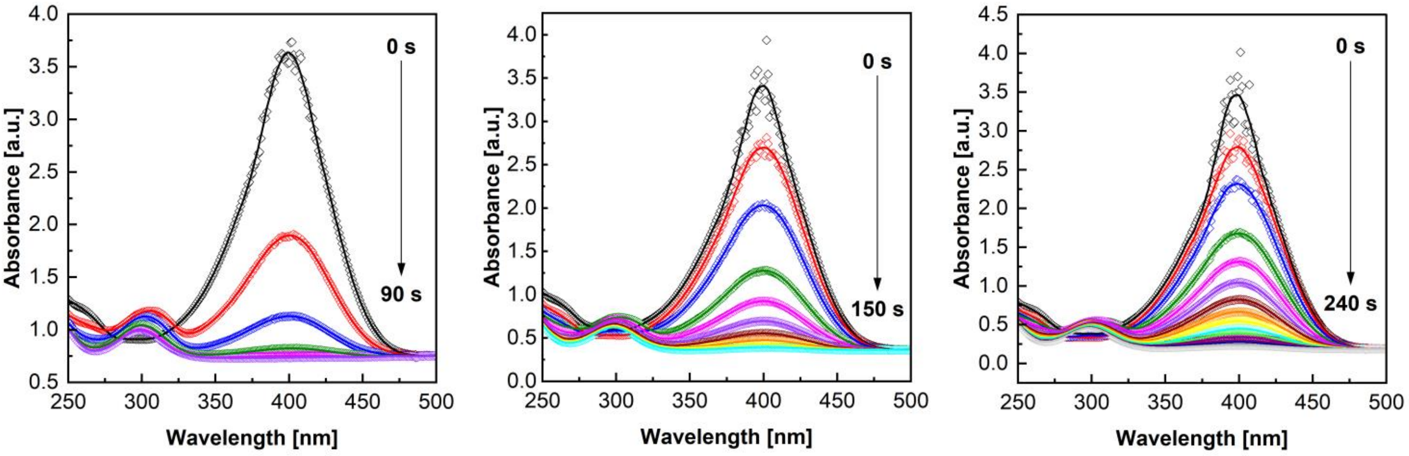

Figure S13. Left to right: reaction monitoring by UV-Vis using conditions **CAT 1-3**. Scatter displays raw data; line represents smoothed data.

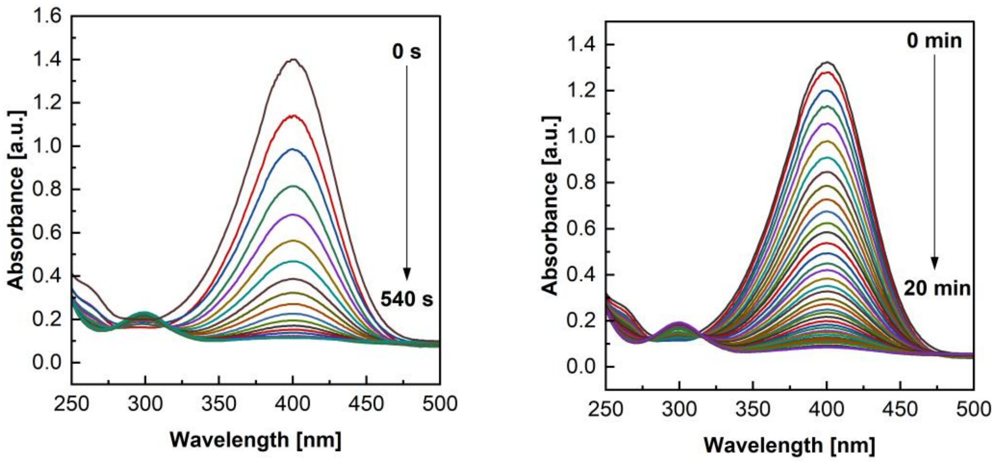

Figure S14. Left to right: reaction monitoring by UV-Vis using conditions **CAT 6-7**.

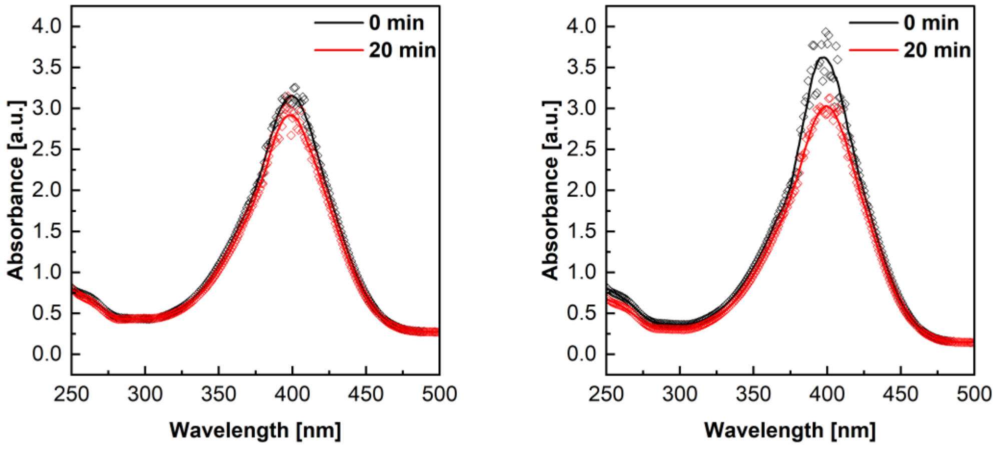

Figure S15. Left to right: reaction monitoring by UV-Vis using conditions **CAT 4-5**. Scatter displays raw data; line represents smoothed data. Reduced absorbance observed in **CAT 5** is attributed to the absorption capabilities of porous HCP networks.

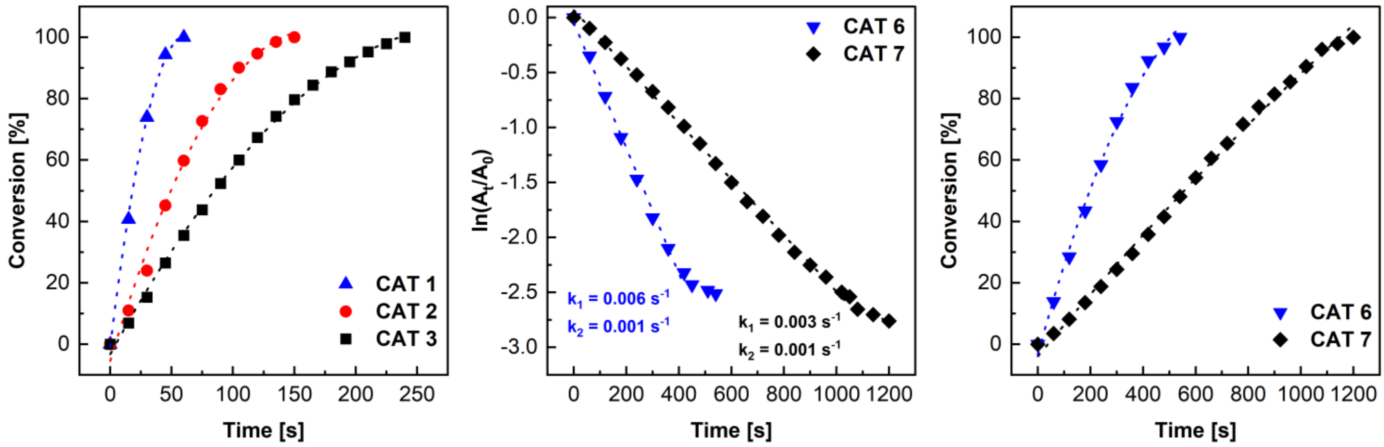

Figure S16. Left: Comparative catalytic conversion by using conditions **CAT 1-3**; middle: kinetics and corresponding rate constants $k^{1/2}$ of conditions **CAT 6-7**; right: Comparative catalytic conversion by using conditions **CAT 6-7**. For clarity every second data point is displayed.

XPS Spectra and TEM Micrograph of Recovered HCP•NHC@AuNP

HCP•NHC@AuNP was recovered from the reaction mixture *via* two consecutive washing steps with acetone followed by centrifugation.

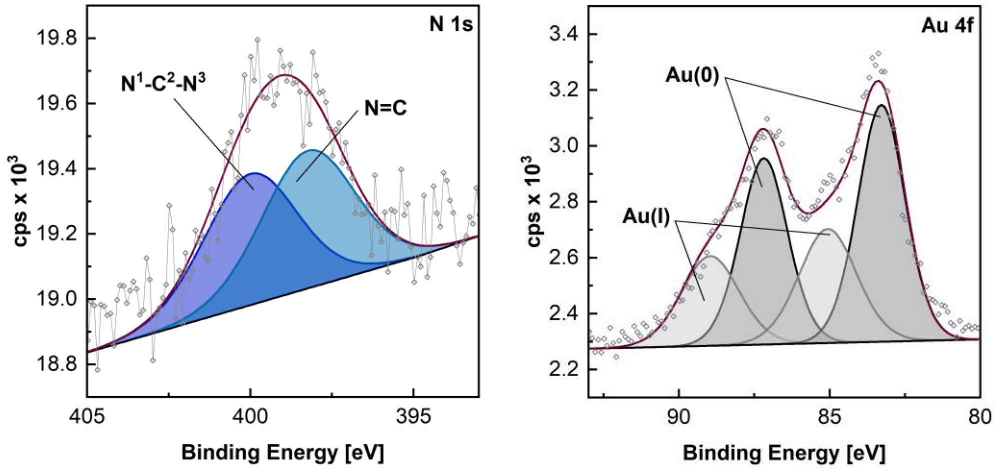

Figure S17. XPS scans of N 1s (left) and Au 4f (right) region of recovered **HCP•NHC@AuNPs** used in conditions **CAT 1**.

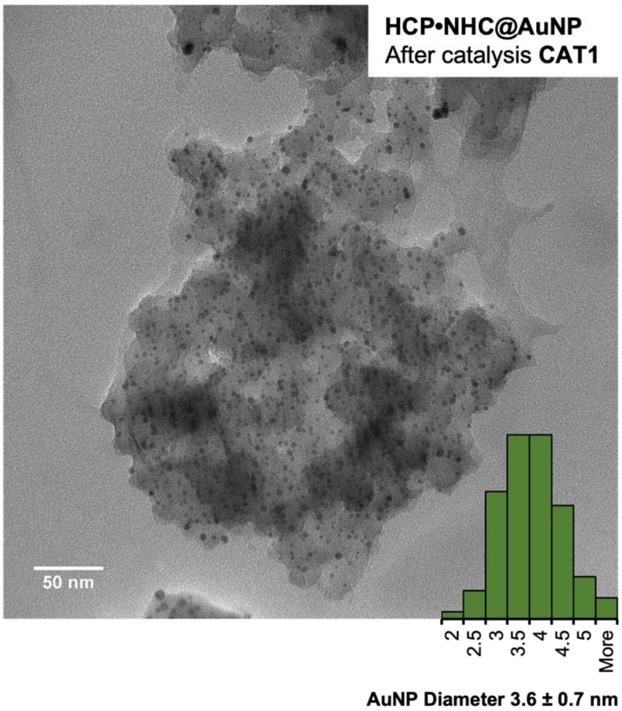

Figure S18. TEM micrograph of recovered **HCP•NHC@AuNP** used in conditions **CAT 1**.

Flow Catalysis

Experiments were carried out in Braun Injekt®-F 1 mL syringes with a compressed pad of **HCP•NHC@AuNP** between two layers of cotton. Flow was provided by a Landgraf Hill LA-30 syringe pump.

Table S5. Conditions of catalytic reduction of 4-NP in flow-through system.

Catalyst	Flow [mL min^{-1}]	Conversion [%][a]	Reaction volume [mL]	HCP_{Cat} Load [mg]	Identifier
**HCP•NHC@AuNP**	0.25	99+	1	6	**FCAT 1**
**HCP•NHC@AuNP**	0.25	60	1	3	**FCAT 2**
**HCP•BIMZ**	0.25	0[b]	1	6	**FCAT 3**
no catalyst	0.25	/	1	/	**FCAT 4**
**HCP•NHC@AuNP**	0.50	99+	1	6	**FCAT 5**
**HCP•NHC@AuNP**	1.0	0[c]	1	6	**FCAT 6**

Conversion of 4-NP^{-} determined *via* UV-vis calibration curve. [b] Absorbance change observed but attributed to chemical absorbance capabilities of **HCP•BIMZ**. [c] Back pressure caused by the hydrophobic **HCP•NHC@AuNP** too high for used experimental setup.

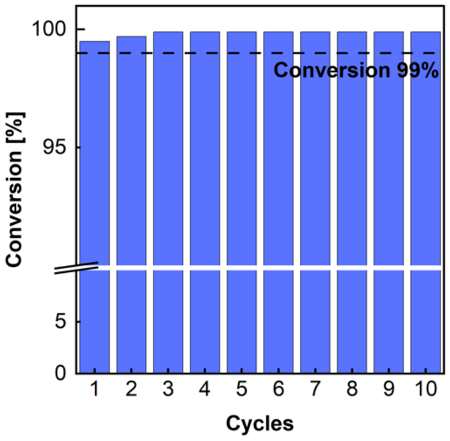

Figure S19. Conversion of 4-NP^- over 10 consecutive cycles in the improvised flow reactor; **FCAT 5**.

XPS Spectra and TEM Micrograph of Recovered HCP•NHC@AuNP from Flow Catalysis

All samples were obtained by careful removal of the **HCP•NHC@AuNP** pad from the syringe enclosure. The **HCP•NHC@AuNP** pad was rinsed with acetone to separate **HCP•NHC@AuNP** and cotton. **HCP•NHC@AuNP** was washed and collected by centrifugation.

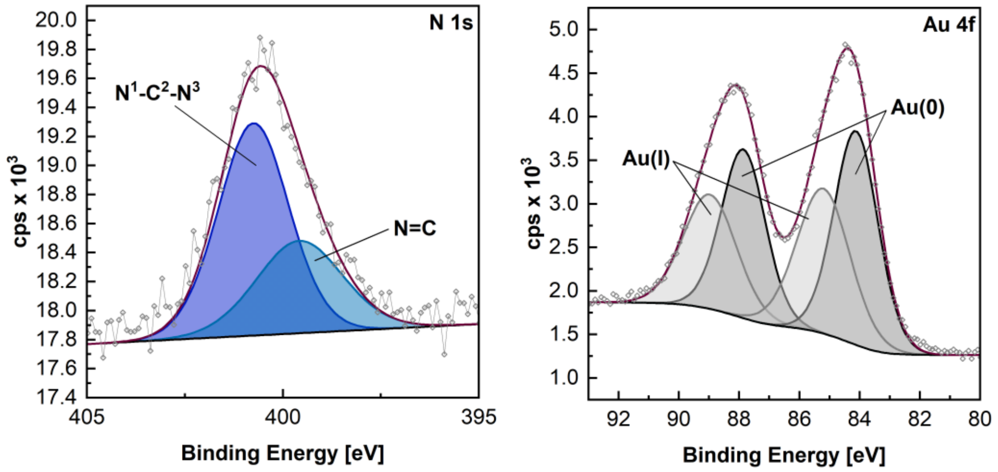

Figure S20. XPS scans of N 1s (left) and Au 4f (right) region of recovered **HCP•NHC@AuNPs** used in conditions **FCAT 1**.

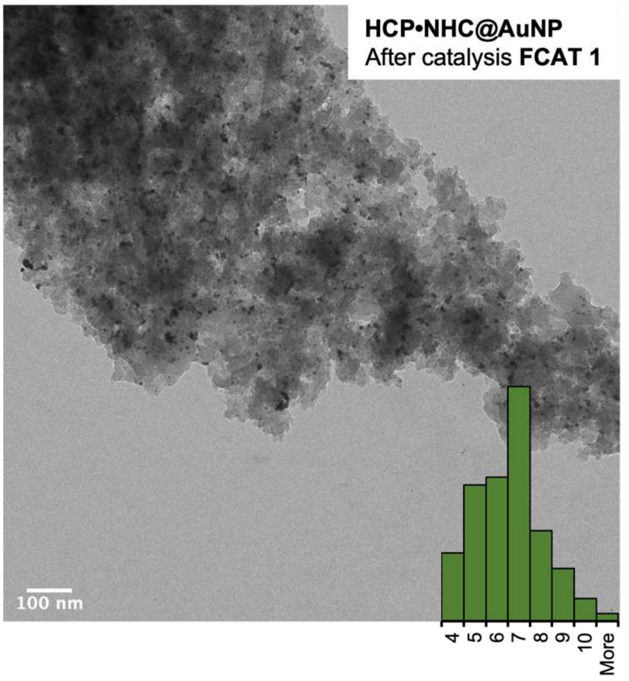

Figure S21. TEM micrograph of recovered **HCP•NHC@AuNP** used in conditions **FCAT 1**.

Comparison of Different Polymer Supported Gold Catalyst for Catalytic Reduction of 4-NP$^-$

Table S6. Conditions for the catalytic reduction of 4-NP.

Reference	AuNP Support	Normalized Rate Constant k_n[min^{-1} μmol^{-1}][a]	wt% Ratio [4-NP/m[Au][b]/$NaBH_4$]	Recyclability
This work	**HCP•NHC***	5.48	1:0.9:421	10 Cycles
He *et al.*[4]	Polystyrene•NHC	49.86	1:0.01:191	/
Nazemi *et al.*[5]	PEG•NHC	4.00	1:0.2:271	/
Tan *et al.*[6]	HCP•Thiol*	0.35	1:0.14:426	8 Cycles
Tan *et al.*[7]	HCP•N-doped	0.80	1:0.4:55	5 Cycles
Banerjee *et al.*[8]	COF*	0.47	1:0.6:426	5 Cycles
Xie *et al.*[9]	COF•Thiol	0.11	1:1.3:146	10 Cycles
Ding *et al.*[10]	COF•Cyclodextrin	3.86	1:0.4:190	7 Cycles

Table displays a sole comparison no ranking. [a] Normalized rate constant calculated according $k_n = \frac{k(min^{-1})}{Au(\mu mol)}$.
[b] Au content on based on published Au wt% of investigated catalyst systems. * System using identical reagent ratios.

References

[1] S. Xu, K. Song, T. Li, B. Tan, *J. Mater. Chem. A* **2015**, *3*, 1272-1278.
[2] L. Prince, P. Guggenberger, E. Santini, F. Kleitz, R. T. Woodward, *Macromolecules* **2021**, *54*, 9217-9222.
[3] a) A. Suwaiyan, R. Zwarich, N. Baig, *J. Raman Spectrosc.* **1990**, *21*, 243-249; b) G. Socrates, *Infrared and Raman Characteristic Group Frequencies: Tables and Charts*, John Wiley & Sons Ltd., Chichester, **2004**.
[4] L. Zhang, Z. Wei, M. Meng, G. Ung, J. He, *J. Mater. Chem. A* **2020**, *8*, 15900-15908.
[5] D. T. H. Nguyen, M. Bélanger-Bouliga, L. R. Shultz, A. Maity, T. Jurca, A. Nazemi, *Chem. Mater.* **2021**, *33*, 9588-9600.
[6] J. He, S. Razzaque, S. Jin, I. Hussain, B. Tan, *ACS Appl. Nano Mater.* **2018**, *2*, 546-553.
[7] L. Tan, B. Tan, *Chem. Eng. J.* **2020**, *390*, 124485.
[8] P. Pachfule, S. Kandambeth, D. Diaz Diaz, R. Banerjee, *Chem. Commun.* **2014**, *50*, 3169-3172.
[9] H. Chen, Q. Zhuang, H. Wang, X. Zhai, K. Zhang, H. Deng, W. Dong, A. Xie, *Colloids Surf., A* **2022**, *649*, 129459.
[10] Y. Zhang, B. Hu, X.-M. Cao, L. Luo, Y. Xiong, Z.-P. Wang, X. Hong, S.-Y. Ding, *Nano Res.* **2020**, *14*, 1018-1025.
